# IgG Expression in Human Colorectal Cancer and Its Relationship to Cancer Cell Behaviors

**DOI:** 10.1371/journal.pone.0047362

**Published:** 2012-11-01

**Authors:** Na Niu, Jie Zhang, Tao Huang, Yingui Sun, Zhengshan Chen, Weining Yi, Christine Korteweg, Juping Wang, Jiang Gu

**Affiliations:** 1 Department of Pathology, Weifang Medical University, Weifang, China; 2 Department of Human Anatomy, Weifang Medical University, Weifang, China; 3 Department of Pathology, Shantou University Medical College, Shantou, China; 4 Department of Anesthesia, Weifang Medical University, Weifang, China; 5 Department of Epidemiology & Biostatistics, Peking University, Beijing, China; University of Aberdeen, United Kingdom

## Abstract

Increasing evidence indicates that various cancer cell types are capable of producing IgG. The exact function of cancer-derived IgG has, however, not been elucidated. Here we demonstrated the expression of IgG genes with V(D)J recombination in 80 cases of colorectal cancers, 4 colon cancer cell lines and a tumor bearing immune deficient mouse model. IgG expression was associated with tumor differentiation, pTNM stage, lymph node involvement and inflammatory infiltration and positively correlated with the expressions of Cyclin D1, NF-κB and PCNA. Furthermore, we investigated the effect of cancer-derived IgG on the malignant behaviors of colorectal cancer cells and showed that blockage of IgG resulted in increased apoptosis and negatively affected the potential for anchor-independent colony formation and cancer cell invasion. These findings suggest that IgG synthesized by colorectal cancer cells is involved in the development and growth of colorectal cancer and blockage of IgG may be a potential therapy in treating this cancer.

## Introduction

Recently several reports have been published describing the expression of immunoglobulins in non-lymphoid cells including several sarcomas and carcinomas [1]–[4]. Prior to these reports, immunoglobulins had always been known to be produced solely by B lymphocytes. IgG has been detected in colon cancer tissue of a few cases and the fragment of IgG constant region has been seen in a couple of colon cancer cell lines. With an antibody against CA215, which was a part of immunoglobulins expressed by ovarian cancer cells, Lee et al detected IgG heavy chain constant regions in colon cancer tissues with a positive ratio of 44% [1], [5]. In 2003, Qiu et al demonstrated expression of IgG in tissues of 6 cases of colorectal cancer (CRC) and in a colon carcinoma cell line (HT29) [3]. With RT-PCR, Kimoto et al detected IgG heavy chain constant region from a colon cancer cell line SW116 [2]. *In vitro* study with a HT 29 colon carcinoma cell line induced with ASODN suggested that cancer-derived IgG suppressed apoptosis [6], which was in keeping with the results of Lee et al [1]and Qiu et al [3] who showed tumor growth inhibition with a carcinoma cell line in animals and in vitro experiments. These observations give rise to the hypothesis that cancer-derived IgG promotes colon cancer growth and this is the focus of the present study.

Thus far, the relationship between IgG expression and clinicopathological features and biological markers [7]–[9] associated with prognosis and response to therapy in CRC has not been investigated. In this study, we first investigated the expression of IgG in tissue of 150 CRC cases and analyzed the correlation of IgG expression and several clinicopathological and biological features. Then IgG production was confirmed in four CRC cell lines at both protein and mRNA levels. Several regions of IgG heavy and light chains and essential enzymes for IgG synthesis were detected in the samples. The effects of IgG on malignant biological behaviors such as proliferation, clone formation, invasion and apoptosis were investigated with experiments of antibody neutralization and siRNA inhibition. The results provide insights into new clinico-pathological roles of cancer-derived IgG in CRC and strengthen the rationale for developing therapies that target cancer-derived IgG.

## Materials and Methods

### Tissue samples

Formalin-fixed, paraffin-embedded tissues from 150 cases that had been treated/analyzed for CRC and the matched normal colorectal mucosa specimens (used as negative controls) and twenty fresh biopsy samples of colorectal adenocarcinoma were collected from the Affiliated Hospital of Weifang Medical University. pTNM stage was made according to TNM staging system of AJCC/UICC [10]. Differentiations of the cancers were graded as well/moderate or poor. Inflammatory infiltration was assessed according to the criteria for chronic inflammation (mononuclear cell infiltration) described previously [11]. The study was approved by the Ethical Committee of Weifang Medical University and written consent was obtained from the patients.

### Cell lines

Human CRC cell lines of different differentiation (moderately differentiated, HT29 and SW480; poorly differentiated, LOVO and HCT116) [12] and Raji (B lymphocytic leukemia cell line as a positive control), Jurkat (T lymphocytic leukemia cell line as a negative control) were purchased from ATCC (June 12, 2008). CRC cell lines were cultured in DMEM with Ultralow-IgG fetal bovine serum (Gibco, Carlsbad, CA, USA) and in DMEM only 12–24 hours before the experiment.

### Immunohistochemistry

Immunohistochemistry (IHC) was performed on CRC and matched marginal tissues with appropriate controls as described previously [13], [14]. Details of primary antibodies to immunoglobulin γ chain (Igγ), immunoglobulin κ chain (Igκ), CEA (carcinoembryonic antigen), CD16 (FcγR III), CD32 (FcγR II), CD64 (FcγR I), P53, PCNA, Bcl-2, MMP-2, NF-κB, and Cyclin D1 are listed in Table S1. Normal goat serum substituted for primary antibody was used as negative controls. Double labeling of IgG and NF-κB or Cyclin D1 or PCNA in cancer cells was performed as described previously [15].

### Scoring immunoreactivity

The stained sections were examined and scored independently by 2 of the authors (N.N and W.Y) without information of the clinicopathological data. Evaluation was performed on five randomly selected cancer regions at a 400× magnification. Levels of IgG expression in cancer cells were based on the sum of the score of the percentage of positive cells (scored as: 0 = <5%, 1 = 5–25%, 2 = 25–50%, 3 = >50%) and that of staining intensity (scored as: 0, absence of signal; 1, light red or pink; 2, red; 3, dark red). The cases with scores of 0–3 were labeled as ‘weakly stained’ and the cases with 4–6 as ‘strongly stained’. Nuclear-located proteins were only scored according to their positive percentage in cancer cells [16]: <25% was grouped as “negative”, ≥25% was grouped as “positive”.

### cRNA probe preparation and in situ hybridization

The appropriate length of probe for ISH was 300–500 bp, and the constant regions of κ and λ chain was too short to be effective probes. Therefore two specific cRNA antisense probes directed against human immunoglobulin G1 heavy chain (IGHG1) were prepared and in situ hybridization (ISH) was performed as described previously [4], [17]. Tonsil tissue and tissues of lymphatic nodules in the colorectal wall were used as the positive control, and tonsil tissue slides incubated with sense probes or CRC tissue incubated with an unrelated probe were employed as negative controls.

### Short tandem repeat analysis

Short tandem repeat (STR) analysis of the CRC cell lines was performed by LAND·HUAGENE BIOSCIENCES CO., LTD (Guangzhou, China) using PowerPlex 16 HS System (Promega).

### Flowcytometry

For detection of IgG, flow cytometry with HT29, SW480, HCT116, LOVO cell lines were performed as described previously [3]. Monoclonal mouse anti-human Igγ (1∶500, Sigma) and FITC-labeled goat anti-mouse IgG (1∶100, Jackson, West Grove, PA, USA) were used as the primary and secondary antibodies. Raji cell line was used as a positive control. FITC-CD19 (Sigma) was used to check for possible contamination by B lymphocytes.

### Immunofluorescence double staining

Immunofluorescence (IF) double staining with Igγ and Igκ was performed on cell lines as described previously [4], [13]. Goat anti-rabbit IgG-TRITC (1∶100, Jackson) or goat anti-mouse IgG-FITC (1∶100, Jackson) was used as the secondary antibody. Normal mouse IgG and normal rabbit IgG (Santa Cruz) were used instead of the primary antibodies. DAPI (blue signal) (1∶500; Sigma) was used to visualize the nuclei.

### RT-PCR

Total RNA was isolated from the cell lines using TRIzol reagent (Invitrogen, Carlsbad, CA, USA). RQ1 RNase-free DNase (Promega, Madison, WI, USA) was used to treat RNA samples to exclude contamination by genomic DNA. Five µg of total RNA extracted was reverse transcribed with oligo (dT)_18_ primer (Fermentas, Burlington, Ontario, Canada) using SuperScript III Reverse Transcriptase (Invitrogen) according to the manufacturer's protocol.

PCR was carried out with LA Taq polymerase (TaKaRa, Dalian, China). Specific primers for IGHG1, the third complementarity determining region (CDR3) of the heavy chain gene (which includes the V-D-J sequence), constant (Igκ) and variable (Vκ) region of Igκ, constant region of Igλ (Igλ), Iγ-Cγ sterile germ line transcripts (Iγ-Cγ), activation-induced cytidine deaminase (AID), recombination activating gene -1 and 2 (RAG1 and RAG2) were the same as described previously [3], [4]. CD19 was amplified to exclude contamination by lymphocytes and β-actin was amplified as the internal reference [3]. Details of the primers were listed in Table S2. Identification of the PCR product was confirmed by DNA sequencing and blasting.

### Laser capture microdissection (LCM) assisted RT-PCR

LCM assisted RT-PCR was performed on 20 cases of freshly frozen surgical CRC samples as described previously [4]. IGHG1, Igκ, CDR3 recombination segment, CD19 and β-actin were amplified with the same primers as for RT-PCRs. Lymphocytes of lymphatic nodules in the colonic wall were employed as a positive control, and water instead of the cDNA template was used as a negative control. Samples with detectable CD19 were excluded from subsequent experiments.

### Western blot and siRNA down-regulation

The cytosol proteins of four human CRC cell lines (40 µg/lane) were analyzed with 10% SDS-PAGE (Igγ under nonreducing conditions and Igκ under reducing conditions). Standard human serum IgG (0.02 µg/lane, Sigma) was used as positive control. Goat anti-human Igγ (1∶2500; Sigma) or the mouse anti-human Igκ (1∶2000, Abcom) were used as the primary antibodies.

SiRNA (5′-CCAAGGACACCCUCAUDAUTT-3′, 5′-AUCAUGAGGGUGUCCUUGGTT-3′) was designed according to the constant region of cancer-derived IgG and transfected into LOVO cell with X-tremeGene SiRNA Transfection Reagent (Roche, Mannheim, Germany) according to the manufacturer's protocol. A random sequence was employed as the negative control. Effectiveness of IgG down-regulating was verified with Western blot.

### Animal studies

Cultured LOVO cells (5×10^6^) were s.c inoculated into the left front armpits of severe combined immunodeficiency (SCID) mice. Fifty µl blood was obtained from the eye vein using capillary tubes at the 0th, 7th, 14th and 21st day after inoculation. 1 µl serum was analyzed with 10% SDS-PAGE under non-reducing conditions with the same antibodies as described above. At day 21, the tumors were harvested and H&E staining, IHC and ISH were performed on tissue sections.

### Cell proliferation and apoptosis analysis

A MTS cell proliferation assay (CellTiter 96 AQ One Solution Cell Proliferation Assay Kit, Promega) was carried out in a 96-well plate according to the manufacturer's instructions. Mouse anti-human Igγ antibody (10–1000 nM, Sigma) was added to serum-free culture medium and incubated for 48 h and OD490 was analyzed [18]. Mouse anti-human IgM antibody (μ chain specific, 10–1000 nM, Sigma) were used as negative control. All experiments were performed in triplicates. Neutralization of IgG antibody with rabbit anti-mouse IgG was performed with increasing concentrations (0–1000 nM) of rabbit anti-mouse IgG added to the supernatant immediately following the addition of 100 nM of mouse anti-human Igγ.

For apoptotic analysis, CRC cells were incubated for 48 h in serum-free culture medium with 100 nM IgG and analyzed with Annexin V-PI kit (Peking University Human Disease Genomics Research Center, Beijing, China) as described previously [3]. IgM antibody (100 nM, Sigma) was used as a negative control. LOVO cells transfected by siRNA was also investigated for proliferative and apoptotic property as described above.

### Soft agar assay on clone formation

Soft agar assay was carried out as described previously [19]. Mouse anti-human Igγ (100 nM, Sigma) was used and anti-human IgM antibody (100 nM, Sigma) or PBS in place of Igγ antibody was used as controls.

### Cell invasion assay

Modified Cell migration assay was performed with LOVO cells and 100 nM mouse anti-human IgG antibody (Sigma) as described previously [20]. The membrane was stained with H&E. Five random fields per membrane were photographed with a BX51 microscope (Olympus) at ×400 magnifications. The invaded cells were counted and average numbers were calculated for each membrane. LOVO cells transfected by siRNA was also examined.

### Statistical analysis

Difference in IgG expression between normal mucosa samples and primary tumors, difference in IgG expression among the various clinicopathological features, and the correlation between IgG expression and the various cancer-related biological variables were tested using the χ^2^ test. Two-sided p values of less than 5% were considered statistically significant. Most calculations were carried out with SPSS 12.0.

## Results

### Expression and distribution of IgG protein and its mRNA in human CRC tissue

**Figure 1:**
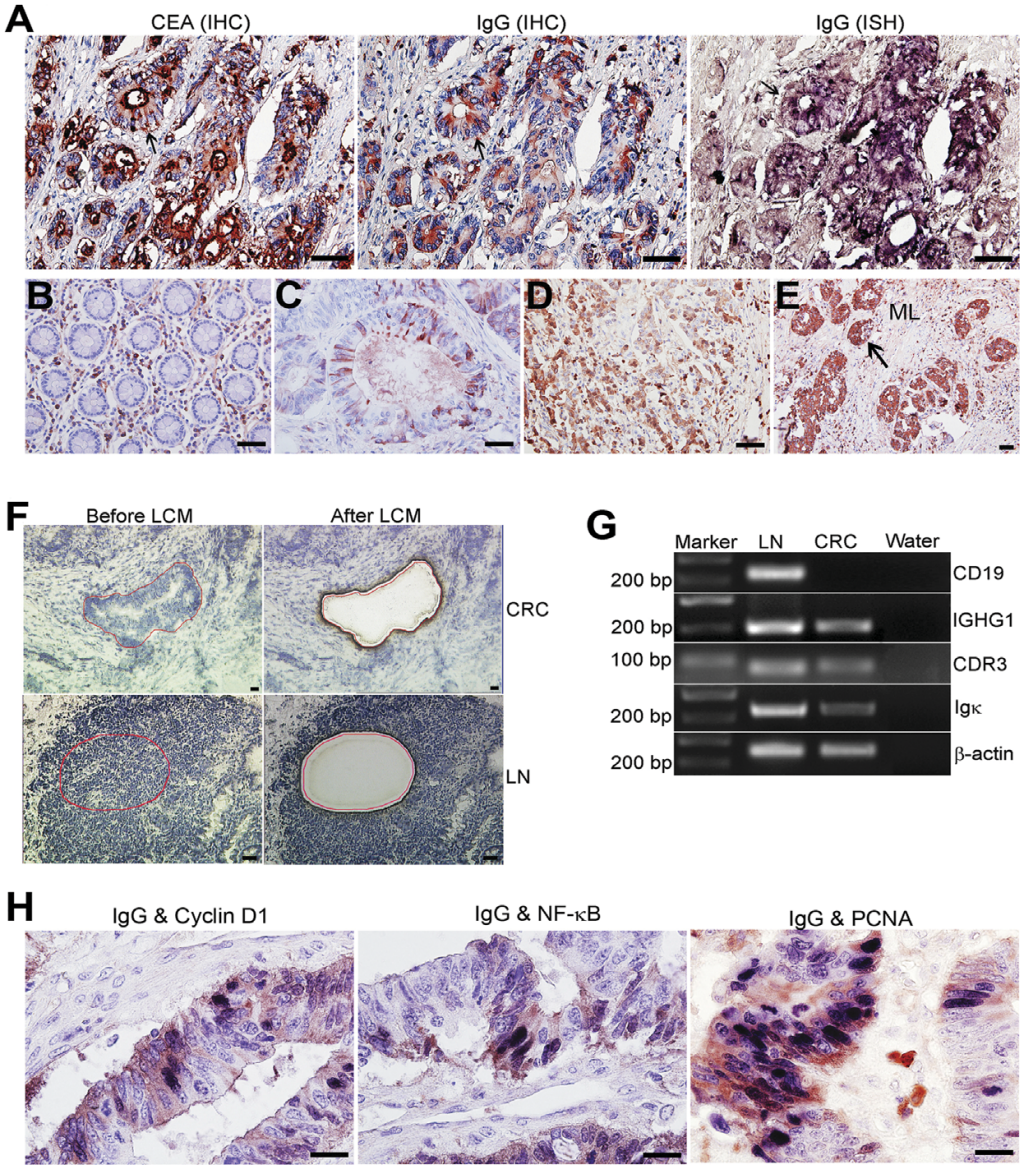
Expression of IgG and mRNA in CRC tissues. **A**: Co-expression of CEA, IgG protein and IGHG1 mRNA in CRC cells. **B–D**: Expression of IgG (red signals) in normal colon tissue (**B**), well differentiated (**C**) and poorly differentiated (**D**) CRC. **E**: Expression of IgG in cells of cancer nests (arrows) infiltrating into deep muscle layer of the colon wall. The expression level of IgG is higher in infiltrating cancer cells (**E**) than in the cancer cells of the “primary” tumor shown in **D**. **F**: CRC nests (CRC) and lymph node (LN) before and after LCM. **G**: Agarose gel electrophoresis of RT-PCR amplification of microdissected CRC cells and lymph nodes. **H**: Co-localization of IgG protein expressed in the cytoplasm (red signal) and Cyclin D1 (**left panel**), NF-κB (**middle panel**) and PCNA with nuclear localization (**right panel**) (purple-blue signals). ML: Muscle layer. Bars = 50 µm (**A–E, G**), 20 µm (**F**).

In all the cases investigated, both Igγ and Igκ were detected mainly in the cytoplasm of cancer cells and B lymphocytes in the stroma (Figure 1A–E), and also in the mesenchymal connective tissue (but not the smooth muscle) of cancer tissues (Figure 1A, E). Co-expression of CEA with IgG and its mRNA was detected on consecutive tissue sections (Figure 1A). In the matched normal mucosa specimens, IgG was not detectable in the glandular epithelium (Figure 1B). Igγ was weakly expressed in 83 (55.3%) and strongly expressed in 67 (44.7%) of the 150 adenocarcinomas. IgG receptors were only detected in the stromal inflammatory cells, but not in the cancer cells.

For adenocarcinoma, the expression of IgG differed among the differentiation grades. Generally, only a few isolated cancer cells (<25%) were found positive in well differentiated adenocarcinomas (Figure 1C), while more single cancer cells or clustered ones were positive in moderately differentiated adenocarcinomas, and most cancer cells (>50%) were positive in poorly differentiated adenocarcinomas (Figure 1D). In the cancer nests infiltrating into the deeper muscular layer, the positive ratio was higher and the staining intensity stronger than in the non-infiltrating cancer cells located in the mucosa (Figure 1E and D).

IgG mRNA (IGHG1) was detectable in the cytoplasm of cancer cells with in situ hybridization (Figure 1A, right panel; Figure S1 A). The specificity of the probes and the stringency for ISH was well established with 2 pairs of probes (antisense and sense) for different regions of IgG constant region and one random unrelated probe. All 2 antisense probes gave completely identitical in distribution and intensity of positive signals. In the positive control, the lymphatic nodules of the colorectal wall (Figure S1 B), IGHG1 was located in the cytoplasm of B lymphocytes. In the negative controls (random probe applied to CRC tissues and sense probes applied to tonsil tissue), no positive signal was detected (Figure S1 C and D). All cases tested positive for IgG immunostaining were also positive for IGHG1 mRNA in situ hybridization. The presence of IgG mRNA was also confirmed with LCM assisted RT-PCR (Figure 1F and G). IGHG1 and Igκ were both successfully amplified following isolation of the cancer cells from frozen CRC samples. All twenty samples tested positively. Moreover, CDR3, including the V-D-J sequence, was also successfully amplified from these samples. No CD19 transcripts were detectable, thereby, excluding B lymphocyte contamination. CD19, IGHG1, Igκ, and CDR3 were all amplified from the positive control samples. No band was detectable when the negative control was used.

### Correlation of IgG expression with clinicopathological and biological features of CRC

Table 1 shows the correlation of IgG expression in primary cancer cells with the various clinicopathological variables. There was a higher frequency of strong IgG expression in poorly differentiated adenocarcinoma (67.3%) than in better differentiated ones (well and moderately differentiated, 33.7%, P = 0.035). IgG expression did not show significant difference among the individual pTNM stages (P = 0.091). However, when we compared the IgG expression in the stage I–II neoplasm with that in the stage III–IV, the latter had a higher frequency of strong staining than did the former (59.7 vs. 32.5%, P = 0.027). When cancers with weak inflammatory infiltration were compared to those with strong inflammatory infiltrate, there was a tendency for the former to have a higher frequency of strong staining than the latter (49.5 vs. 33.3%, P = 0.041). In term of lymph node staging, CRC of stage N_1_ and N_2_ occupied a significantly higher IgG-positive ratio (54.8, 63.9 vs 32.5%, P = 0.042). There was no significant correlation of IgG expression with age, gender, cancer location or growth pattern (all P>0.05).

**Table 1:** Correlations Between IgG Expression and Various Clinicopathological Features of CRC.

Variable	n	IgG expression		P value^a^
		Weakly stained (0–3)	Strongly stained (4–6)	
Age, years				0.843
≤40	23	13 (56.5)	10 (43.5)	
>40	127	70 (55.1)	23 (41.9)	
Gender				0.771
Male	82	45 (54.9)	37 (45.1)	
Female	68	38 (55.9)	30 (44.1)	
Tumor location				0.945
Colon	66	37 (56.1)	29 (43.9)	
Rectum	84	46 (54.8)	38 (45.2)	
Growth pattern				0.295
Expansive	32	18 (56.3)	14 (43.7)	
Infiltrative	118	65 (55.1)	53 (44.9)	
Differentiation				0.035
Well/moderate	101	67 (66.3)	34 (33.7)	
Poor	49	16 (32.7)	33 (67.3)	
pTNM stage				
I	24	16 (66.7)	8 (33.3)	0.027^b^
II	59	40 (67.8)	19 (32.2)	0.174^c^
III	54	22 (40.7)	32 (59.3)	0.096^d^
IV	13	5 (38.5)	8 (61.5)	
Lymph node stage				
N_0_	83	56 (67.5)	27 (32.5)	0.042^e^
N_1_(1–3)	31	14 (45.2)	17 (54.8)	0.143^f^
N_2_(>4)	36	13 (36.1)	23 (63.9)	
Inflammatory infiltration				
Mild/moderate	105	53 (50.5)	52 (49.5)	0.041
Marked	45	30 (66.7)	15 (33.3)	

Figures in parentheses are percentages.

a χ^2^ test.

b I+II vs III+IV;

c I vs II+III+IV;

d I+II+III vs IV;

e N_0_ vs N_1_+N_2_;

f N_0_+N_1_ vs N_2_.

Table 2 presents the relationship between IgG expression and the expressions of several biological markers. IgG expression showed positive correlation with expressions of Cyclin D1 (P = 0.046), NF-κB (P = 0.019), and PCNA (P = 0.037), and negative correlation with expression of Bcl-2 (P = 0.041), and no significant correlation with the expressions of MMP-2 and p53 (both P>0.05). Co-localization of Cyclin D1, NF-κB or PCNA (nuclear staining) and IgG (cytoplasmic staining) was clearly established with double-immunostaining (Figure 1H).

**Table 2:** Relationships Between IgG Expression and Various Biological Factors of CRC.

Variable	n	IgG expression		P value^a^
		Weakly stained (0–3)	Strongly stained (4–6)	
Bcl-2				
Negative	86	39 (45.3)	25 (54.7)	0.041
Positive	64	44 (68.8)	8 (31.2)	
Cyclin D1				
Negative	58	41 (70.6)	17 (29.4)	0.046
Positive	92	42 (45.7)	50 (54.3)	
MMP-2				
Negative	106	59 (55.7)	47 (44.3)	0.679
Positive	44	24 (54.5)	9 (45.5)	
NF-κB				
Negative	101	65 (64.4)	36 (35.6)	0.019
Positive	49	18 (36.7)	31 (63.3)	
P53				
Negative	110	65 (59.1)	45 (40.9)	0.601
Positive	40	18 (45.0)	22 (55.0)	
PCNA				
Negative	73	51 (69.9)	12 (30.1)	0.037
Positive	77	32 (41.6)	45 (58.4)	

Figures in parentheses are percentages.

a χ^2^ test.

### Expression of IgG protein and its mRNA in CRC cell lines

**Figure 2:**
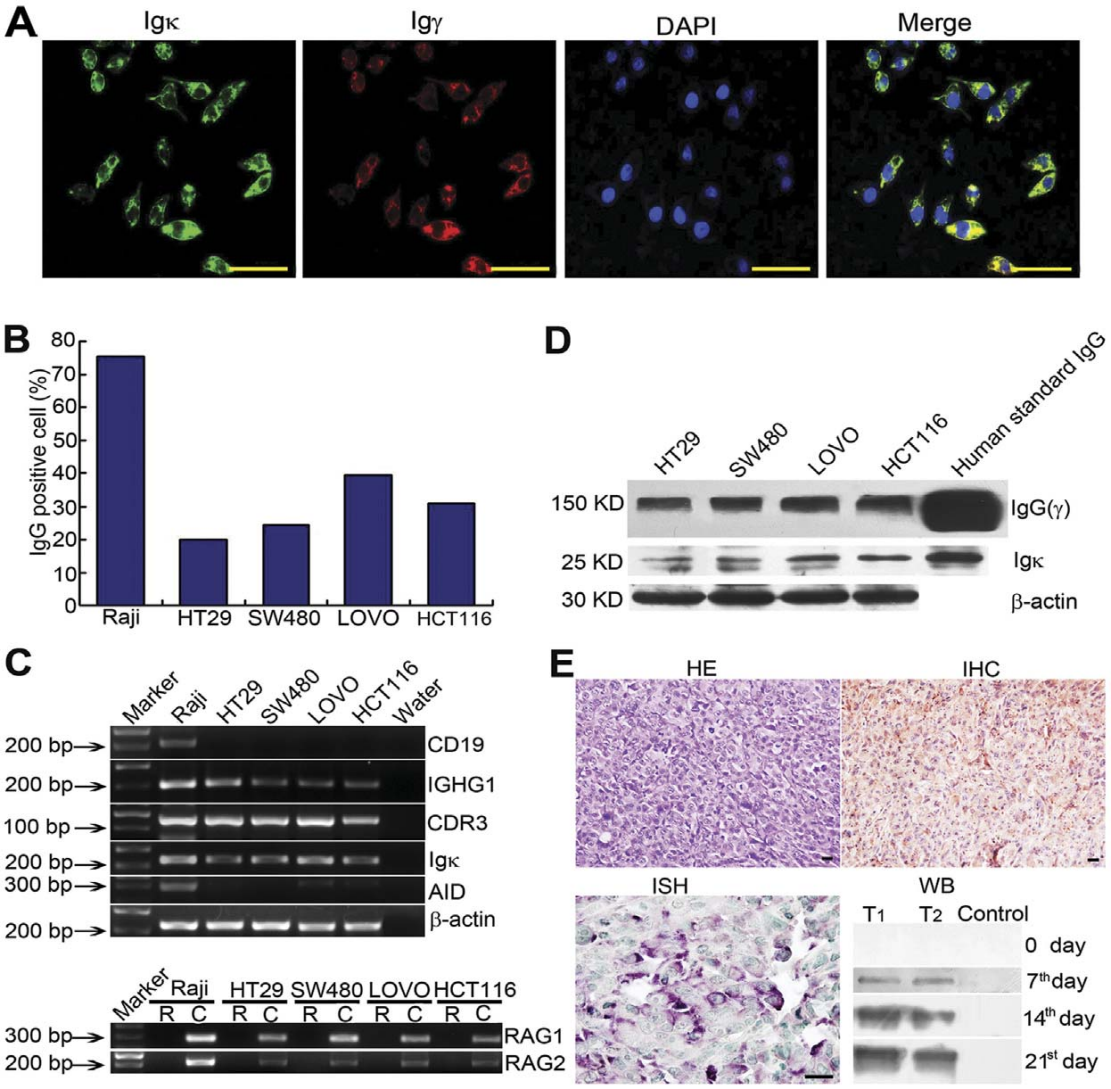
Expression of IgG protein and mRNA in human CRC cell lines. **A**: Igγ and Igκ are detected in all four CRC lines by Double staining immunofluorescence (Igγ, TRITC; Igκ, FITC; Merge, yellow.). Bars = 50 µm. **B**: Percentage of IgG positive cells in Raji, HT29, SW480, and LOVO. **C**: Agarose gel electrophoresis of RT-PCR amplification products. R, DNase treated RNA as template; C, cDNA as template. **D**: IgG detection by Western blot in all four cell lines. **E**: IgG protein and mRNA are detected with IHC and ISH in tumors harvested on 21 days of LOVO cells in SCID mice. IgG is detectable in the sera at day 7, 14, and 21.

The identities of employed CRC cell lines were verified with STR test and the profiles showed 100% confirmation with those provided by ATCC (Figure S2 A and B). Concentration of IgG in Ultralow-IgG FBS for cell culture was confirmed to be 2.3±0.9 µg/ml, sufficiently low for the experiment. IgG protein expression was demonstrated in all four CRC cell lines with IF. Co-expression of Igγ and Igκ was also confirmed (Figure 2A). Igγ and Igκ were mainly localized to the cytoplasm, and partially in the nuclei of cancer cells. No IgG signal was detected in Jurkat cells.

Contamination of B lymphocytes was ruled out using FITC labeled immunostaining of CD19 (Figure S2 C). The percentage of IgG positive cells was determined by FACS with Igγ antibody. The percentage of IgG positive cells was 75.2%, 20.04%, 24.4%, 39.54%, and 30.91% in Raji, HT29, SW480, LOVO and HCT116 cell lines respectively (Figure 2B). The average IgG-positive cell percentage of moderately differentiated HT29 and SW480 cell lines (22.22%) was lower than that of poorly differentiated LOVO and HCT116 cell lines (35.23%, P = 0.043).

In RT-PCR, sensitivity and specificity were well established in several experiments. First, RNase-free DNase was used to eliminate the possible effect of genomic DNA. CD19 was amplified to rule out the possible contamination of B lymphocyte. Second, several regions of IgG heavy and light chains and essential enzymes for IgG synthesis were investigated. Third, extensive positive and negative controls were employed for the same amplification. Forth, all amplified products were sequenced and the identitied of the amplified products were confirmed. Using RT-PCR, IGHG1, Igκ, Vκ, Igλ and Iγ-Cγ transcripts were detected in all four cell lines, with the intensities of the positive bands being weaker than those of the Raji cells. Moreover, CDR3, including the V(D)J recombination sequence and RAG1 and RAG2 were also detected, whereas AID was detected only in LOVO and HCT116 cell lines (Figure 2C and Figure S1E).

Forty (40) µg of total protein was extracted from each cell line. The total protein was run on a Western blot gel and reacted with antibody to IgG. A band at the same molecular weight to, but with less intensity than, the band obtained with 0.02 µg of human IgG was detected (Figure 2D). The better the cell lines were differentiated, the less IgG was expressed. Under reduced condition, Igκ bands were detected at 25 KD in HT29, SW480, LOVO and HCT116 cell lines. Similar to observations by Huang et al [21], we also detected an additional band at 23 KD in HT29, SW480, and LOVO cell lines. The amounts of Igκ were less than those of standard serum IgG.

### Expression and secretion of IgG in tumor bearing SCID mice

At day 21 after inoculation of cultured LOVO cells into the SCID mice, the tumors were harvested. The tumor consisted of clustered cancer cells with indistinct cell borders without stroma. All cancer cells were IgG positive, with granular positive signals in the cytoplasm. The distribution of IGHG1 mRNA detected with in situ hybridization was similar to that of IgG protein detected by IHC (Figure 2E).

SDS-PAGE showed that as the cancer grew larger, significantly stronger bands of human IgG were detected in the sera of SCID mice (Figure 2E).

### Blockade of cancer-derived IgG affects the biological behaviors of CRC cells

**Figure 3:**
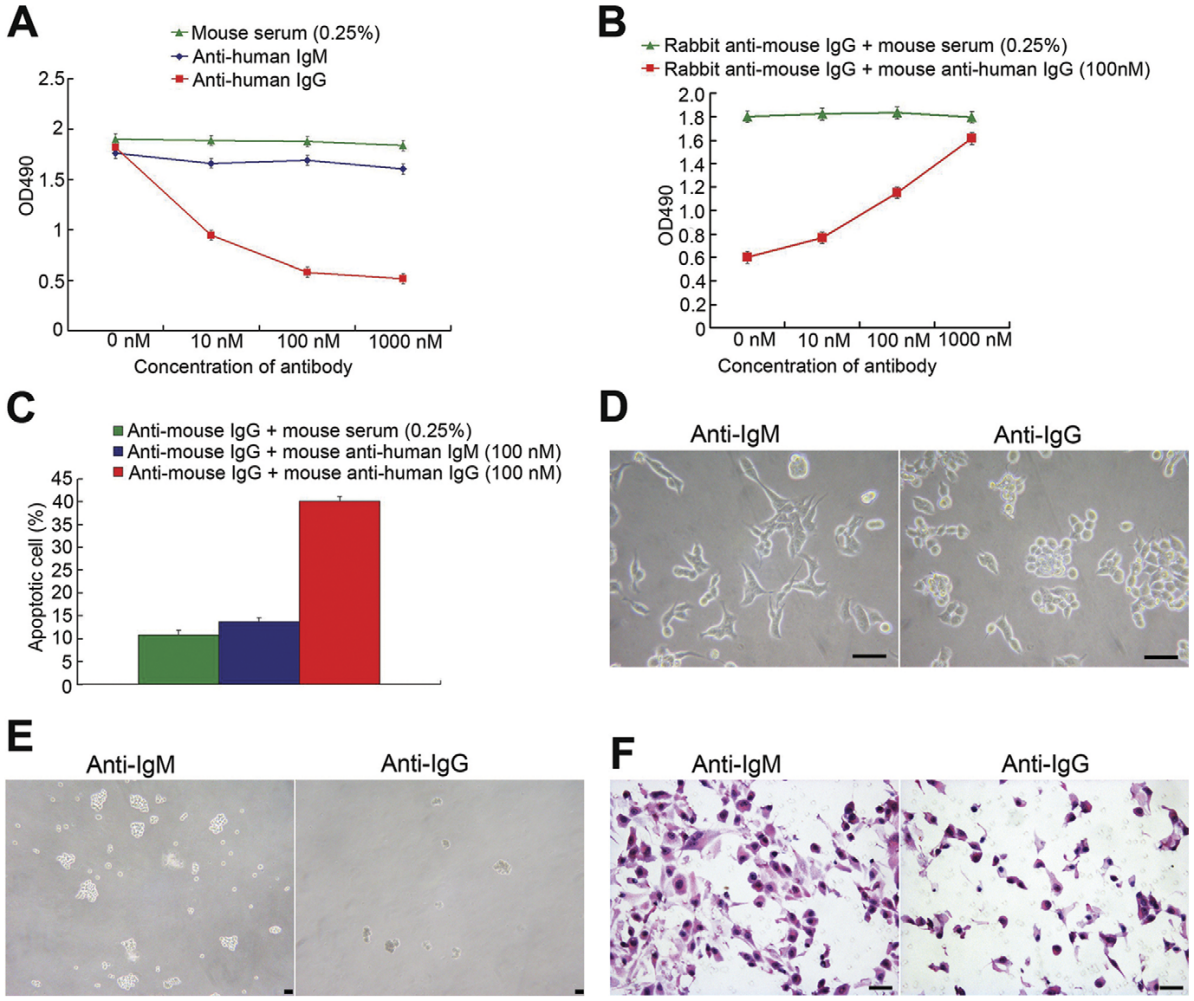
Blockage of cancer-derived IgG by anti-human IgG antibody inhibits biological behaviors of CRC cells. **A**: MTS with a series of concentrations of anti-human IgG (10–1000 nM) antibody. **B**: Treatment with rabbit anti-mouse IgG increases the apoptosis rates in LOVO cells. **C**: Apoptotic percentage of LOVO cells is higher after incubation with IgG antibody than with IgM antibody or mouse serum. **D**: Blockage of cancer-derived IgG results in morphological changes. **E**: IgG antibody decreases malignant anchor-independent growth of CRC cells. **F**: Blockade of cancer-derived IgG suppresses the invasion potential of CRC cells. Bars = 50 µm.

Compared with the negative controls (mouse serum and anti-human IgM), anti-human IgG showed significantly increased inhibition of proliferation of LOVO cells, especially at the 100 and 1000 nM concentrations (Figure 3A). For the ensuing experiment, we chose 100 nM as the most effective concentration. As rabbit anti-mouse IgG can impede the binding of mouse anti-human IgG to human IgG molecules as a result of sterical hindrance [22], it was be used to antagonize the effects of anti-human IgG. Treatment with rabbit anti-mouse IgG effectively abated the inhibition of cancer cell growth by mouse anti-human IgG (Figure 3B).

The apoptosis analysis (Figure 3C) showed that treatment with IgG antibody resulted in an higher rate of apoptotic LOVO cells (40.16%) compared to those treated with IgM antibody (13.7%, P = 0.0364) or mouse serum (10.9%, P = 0.0417).

Morphological features of LOVO cells were also affected by treatment with IgG antibody (Figure 3D). When incubated with anti-IgM antibody (100 nM), LOVO cells grew loosely while developing several cell processes. In contrast, incubated with anti-IgG antibody (100 nM), LOVO cells became round with much less number of cell processes and showed a limited cluster-oriented growth pattern.

Soft agar analysis showed that LOVO cancer cells co-cultured with anti-human IgG antibody grew more slowly and generated significantly smaller colonies, that were not transparent and had blurry cell borders, than those of the same LOVO cancer cells treated with anti-human IgM antibody (Figure 3E). Treatment with PBS instead of IgG antibody gave similar results to that treated with anti-human IgM antibody. The average number of colony formation in LOVO cells treated with anti-human IgG (23.7±5.2/35 mm plane) was significantly smaller than that of LOVO cells treated with IgM antibody (46.1±3.2/35 mm plane, P = 0.0374).

The cell invasion assay demonstrated that the average number of invaded cells was lower in the LOVO cells incubated with anti-human IgG antibody (32.3±3.9) than that in those incubated with IgM antibody (116.5±5.2, P = 0.0293) (Figure 3F).

### Effect of IgG interference on biological behaviors of CRC cells

To investigate possible roles of IgG in biological behaviors further, down regulation of IgG expression with siRNA interference was performed. As shown in Figure S3 A, more than 50% IgG protein synthesis was reduced effectively with siRNA. In MTS, IgG down-regulation suppressed proliferation of LOVO cells (Figure S3 B). Annexin V-PI immunofluorescence double staining showed that early (Annexin V+) and late (PI+) apoptotic cells were increased with IgG knockdown by siRNA as compared to si-control (Figure S3 C). Meanwhile, IgG knockdown also decreased transwell cells (Figure S3 D).

## Discussion

Increased serum levels of IgG and IgA are often detected in patients with epithelial cancers [23], [24] including those with CRC [25], [26]. In addition, IgG and IgA have been found to be expressed in cell lines and tissues of various epithelial cancer types, including CRC [3], [4], [6], [27], [28]. Although the phenomenon of Ig expression in cancers has become established, the function of cancer-derived Ig remains unclear. In addition, little is known about the relationships between cancer-derived IgG and the clinicopathological features and biological behaviors of carcinomas. Chen et al. have recently reported that the expression of IgG in soft tissue sacomas was associated with tumor grade and expression of PCNA, Ki-67 and Cyclin D1 [29]. In the present study, we confirmed the presence of IgG expression in colorectal cells at both the protein and the mRNA levels. The lack of immunoglobulin receptor expression and the detection of IgG mRNA in CRC cells ruled out the possibility that circulating IgG was taken up by these cancer cells. We demonstrated a positive correlation between IgG expression, tumor differentiation grade, pTNM stage and lymph node involvement. Expression of IgG was stronger in CRC tissues with poor differentiation or with pTNM stage III–IV, than in those with better differentiation or pTNM I–II. IgG expression might be negatively associated with inflammatory infiltration, as cancers with lighter inflammatory infiltration showed stronger IgG expression. Our findings in colorectal cancer tissues are consistent with our observations in colorectal cell lines, showing a lower quantity of IgG in cancer cells of moderate differentiation (HT29 and SW480) when compared with those of poor differentiation (LOVO and HCT116). Taken together, these findings suggest that cancer-derived IgG might be involved in the process of de-differentiation, infiltration and metastasis of CRCs, and that it might play a self-protecting role during cancer development. In fact, under certain conditions immunoglobulins have been found to promote cancer growth [30], [31], and this phenomenon has mainly been attributed to the shielding of target epitopes on the surface of cancer cells by “anti-tumor” antibodies [32], [33]. One study of patients with ovarian cancer suggested that such blocking antibodies might be aberrantly glycosylated IgG molecules, which are incapable of eliciting immune responses [24]. Besides immunoglobulin heavy chains, T-cell receptors (such as TCR-a and TCR-b), immunoglobulin -like cell adhesion molecules (such as CD47, CD54, CD58) were also found to be expressed in cancer cells [34]. TCR and IgG antibodies led to complement-dependent cytotoxicity and apoptosis of cancer cell lines. So these “immunoglobulin superfamily proteins” were hypothesized to be self-protective and involved in the proliferation of cancer cells [34]. An alternative, indirect role for IgG in stimulating tumor growth could involve transforming growth factor beta (TGF-β) which is carried by immunoglobulins and has been demonstrated to prevent cytolytic T cell responses [35]. Whether IgG produced by colon cancer cells is aberrantly glycosylated or associated with TGF-β warrants further investigation.

We also studied the relationship between the expression of IgG and that of various biological and prognostic markers in CRC cells, including Bcl-2 (anti-apoptotic protein), Cyclin D1 (proliferation marker), MMP-2 (invasion marker), NF-κB (anti-apoptotic factor and nuclear transcription factor), P53 (tumor suppression gene) and PCNA (proliferation marker). In several studies, over-expression (Cyclin D1, NF-κB, PCNA) or loss of expression (Bcl-2) of these markers have been found to correlate with more aggressive tumor growth in CRC [7]–[9]. P53 is an extensively studied tumor suppression gene and mutations of P53 occurred in more than 50% of sporadic CRC. The phenotype of P53 negative/Bcl-2 positive is thought to be an independent indicator of good prognosis in CRC [36]. PCNA is a well established marker for proliferation of CRC [9]. In the present study, IgG expression was found to be positively correlated to expression of Cyclin D1, NF-κB and PCNA and negatively correlated to expression of Bcl-2. These correlations give additional support to the notion that IgG might be involved in cancer growth and differentiation. MMPs are degradative enzymes important for tumor progression and metastasis [37]. Over-expression of MMPs in CRC indicated a poor prognosis and different expressions of MMP-2 demonstrated significantly different prognoses between Dukes C1 and C2 groups [38], [39]. In the present study, relationship of IgG and MMP-2 expression was analyzed but no significant correlation was found, indicating that IgG may be involved in the progression of CRC independent of MMPs.

A growth promoting role of cancer-derived IgG has previously been suggested by other groups based on their findings that blockade of IgG by either antisense DNA or anti-human IgG antibody increased apoptosis and inhibited growth of HeLa cervical carcinoma and HT 29 colon carcinoma cells *in vitro* [3], [6], and that injection of anti-IgG antibody into HELA cell tumors in nude mice resulted in significantly decreased tumor size compared with untreated nude mice [3]. In this study we further defined the effect of IgG on biological behavior of cancer cells using monoclonal antibodies to human IgG. We first confirmed the apoptosis-suppressing effect of IgG in CRC cells. We then demonstrated that blockage of IgG resulted in morphological changes of cancer cells and that it decreased the ability of clone formation and invasion potential of CRC cells. We also found cancer-derived IgG capable of promoting anchor-independent growth and infiltration. These findings could explain the differences of IgG expression between CRC tissues of pTNM stage I–II and III–IV, and between those of well/moderate and poor differentiations.

We demonstrated, using a SCID mouse model, that tumors can secrete endogenously produced IgG into circulation. Following inoculation of LOVO cells into SCID mice, IgG protein (and mRNA) was not only detected in the harvested tumor cells at day 21, but also in the sera with increasing IgG levels as the tumor grew larger. Weak positive IHC signals were also observed in the stroma of CRC tissues. It is conceivable that IgG, upon production and secretion by cancer cells, regulates proliferation of such cells through autocrine signaling. This notion is raised by analogy to the action of other factors promoting proliferation and invasion that have been identified together with their corresponding receptors in cancers, including epidermal growth factor (EGF), vascular endothelial growth factor (VEGF), and insulin-like growth factor (IGF). However, as no FcγRs were found expressed in CRC tissues, the exact interaction between IgG and cancer cells is unclear.

At the mRNA level, IGHG1 and Igκ were detected in all four cell lines and in the microdissected CRC cells. CDR3 transcripts, including the V(D)J recombination segment, were also detected and confirmed by sequencing. RAG1 and RAG2, the essential enzymes for V(D)J recombination, were also detected in cancer samples, whereas AID, an enzyme required for class switch and somatic hypermutation, was only detected in the two poorly differentiated cell lines (LOVO and HCT116). In agreement with Lee et al [5], our in vivo and in vitro results indicated that IgG derived from CRC cells could be secreted and membrane-bound.

In this study we had to control for the fact that B lymphocytes and plasma cells are widely distributed throughout the gastro-intestinal tract in Peyer's patches, and diffusely in the lamina propria and epithelial cell lining, collectively known as the gut associated lymphoid tissue [40]. To ascertain that IgG was in fact produced by cancer cells but not (only) by resident or infiltrating B lymphocytes various experiments were performed, including LCM combined with RT-PCR, and inoculation of CRC cancer cells in a mouse model deficient of B and T lymphocytes.

In conclusion, IgG was found to be extensively produced and secreted by CRC cells. The expression level of IgG was correlated with prognostic clinicopathological characters, including differentiation, pTNM stage, and expression of various biological markers. Cancer-derived IgG can promote growth, clone formation and invasion of the neoplastic cells. These results suggest that cancer-derived IgG might be involved in the process of carcinogenesis, neoplastic infiltration and regulation of CRCs. As IgG production in various neoplastic cells is likely a common phenomenon, the significance of locally produced IgG in regulating the behavior of cancers in evident, and the mechanism of which warrants further investigation.

## Supporting Information

Figure S1 **In situ hybridization (ISH) and RT-PCR analysis of IgG in CRC.** **A**: ISH in which antisense probe for constant region of IgG was applied. **B**: Positive control (lymphatic nodules of the colorectal wall) of ISH of IgG. **C, D**: ISH results in which random probe applied to CRC tissues (**C**) and sense probes applied to tonsil tissue (**D**). Bars, 50 µm. **E**: mRNA expression of Vκ, Igλ and Iγ-Cγ by RT-PCR in four CRC cells.
(PDF)

Figure S2 **Confirmation of colorectal cancer cell lines.** **A, B**: STR profiles of the colon cancer cell line HCT116 (**A**) and SW480 (**B**). **C**: Signals of CD19 (Green) were detected with flow cytometry in Raji cell, but not in CRC cell lines.
(PDF)

Figure S3 **Reduction of IgG expression by siRNA affects the biologic behaviors of LOVO cells.** **A**: Effect of IgG down-regulation. **B**: MTS assay shows that IgG down-regulation inhibits growth of LOVO cells. **C**: AnnexinV-PI Immunofluorescence double staining analysis shows that the apoptotic LOVO cells when IgG was downregulation, nuclear staining with DAPI. **D**: Invasion assay was carried out with transwell in 24 wells when si-IgG transfected into LOVO cells for 24 hours.
(PDF)

Table S1 **Primary Antibody Details.**
(PDF)

Table S2 **Primers for PCR Amplification.**
(PDF)
