# Supplementary material for: IgG Expression in Human Colorectal Cancer and Its Relationship to Cancer Cell Behaviors
Source: PLoS One. 2012 Nov 1;7(11):e47362. doi: 10.1371/journal.pone.0047362 (PMC3486799; doi:10.1371/journal.pone.0047362)
Supplement: Figure S1 — In situ hybridization (ISH) and RT-PCR analysis of IgG in CRC. A: ISH in which antisense probe for constant region of IgG was applied. B: Positive control (lymphatic nodules of the colorectal wall) of ISH of IgG. C, D: ISH results in which random probe applied to CRC tissues (C) and sense probes applied to tonsil tissue (D). Bars, 50 µm. E: mRNA expression of Vκ, Igλ and Iγ-Cγ by RT-PCR in four CRC cells. (PDF) [file pone.0047362.s001.pdf]

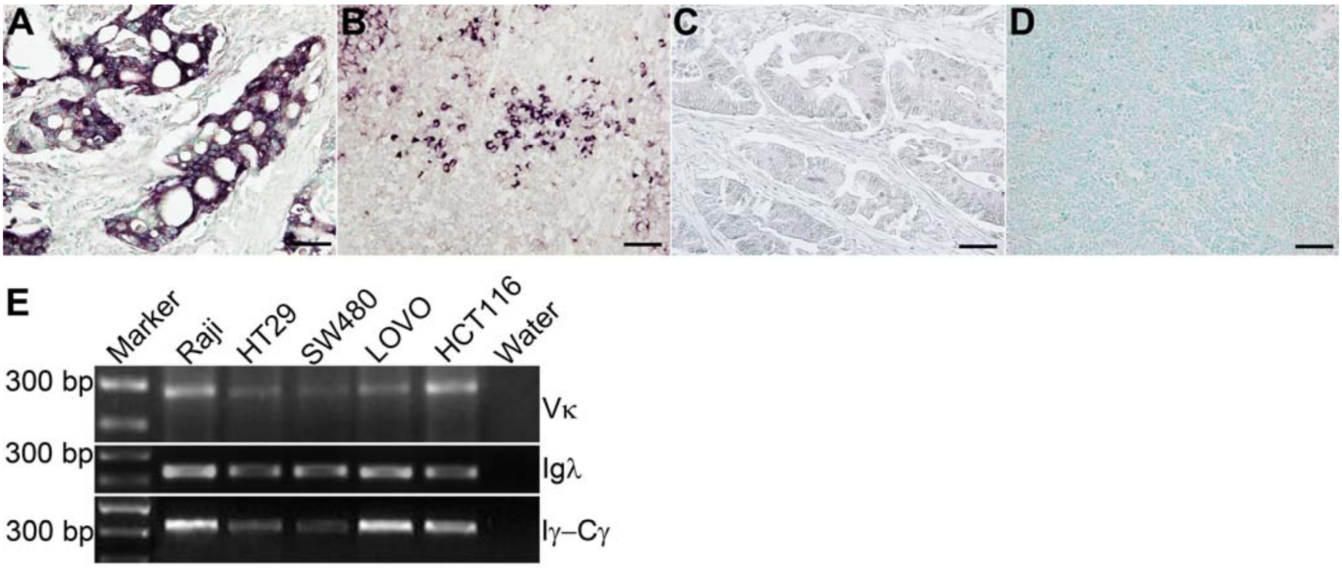

Figure S1. In situ hybridization (ISH) and RT-PCR analysis of IgG in CRC. **A:** ISH in which antisense probe for constant region of IgG was applied. **B:** Positive control (lymphatic nodules of the colorectal wall) of ISH of IgG. **C, D:** ISH results in which random probe applied to CRC tissues (**C**) and sense probes applied to tonsil tissue (**D**). Bars, 50μm. **E:** mRNA expression of Vκ, Igλ and Iγ-Cγ by RT-PCR in four CRC cells.
