# Supplementary material for: IgG Expression in Human Colorectal Cancer and Its Relationship to Cancer Cell Behaviors
Source: PLoS One. 2012 Nov 1;7(11):e47362. doi: 10.1371/journal.pone.0047362 (PMC3486799; doi:10.1371/journal.pone.0047362)
Supplement: Figure S2 — Confirmation of colorectal cancer cell lines. A, B: STR profiles of the colon cancer cell line HCT116 (A) and SW480 (B). C: Signals of CD19 (Green) were detected with flow cytometry in Raji cell, but not in CRC cell lines. (PDF) [file pone.0047362.s002.pdf]

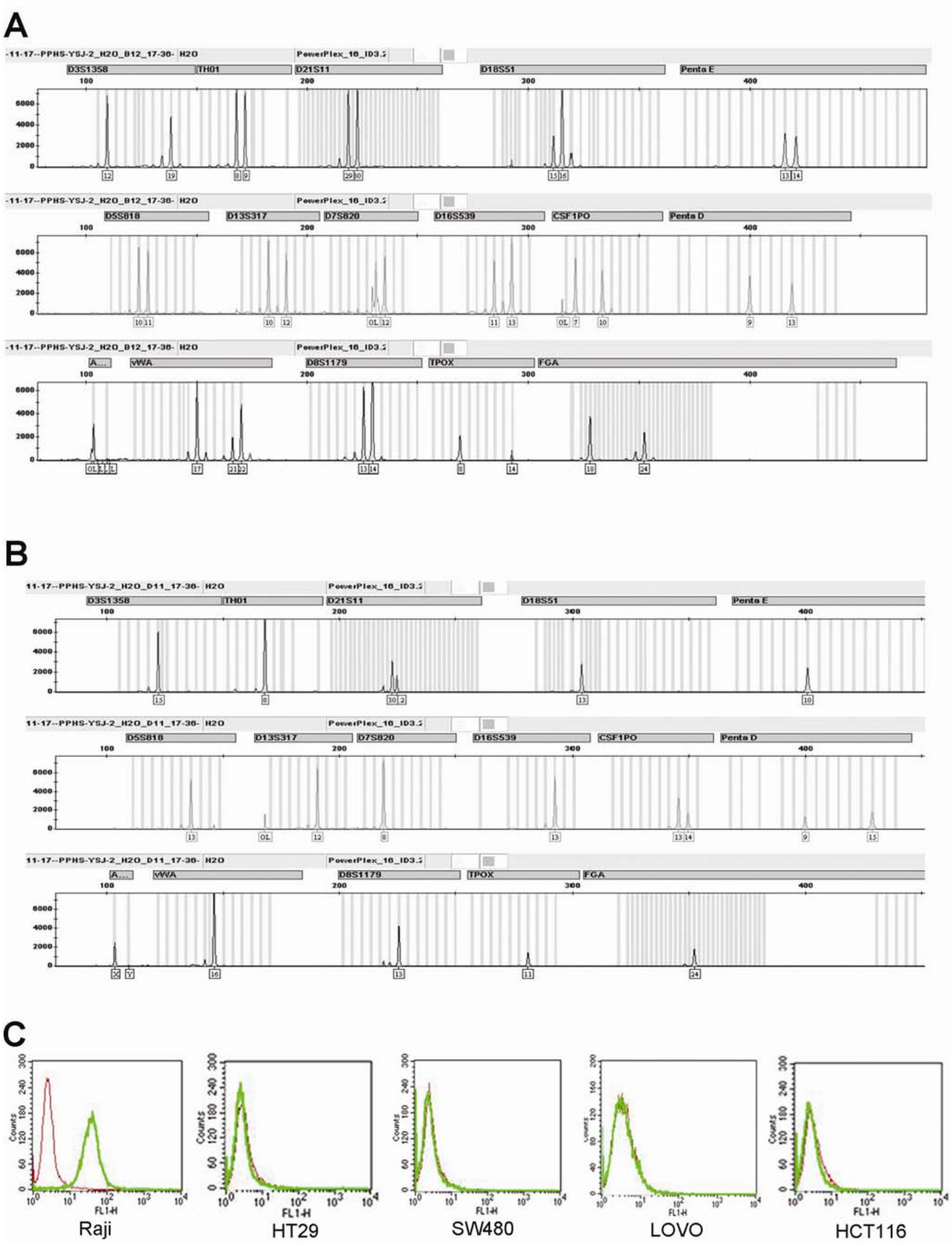

Figure S2. Confirmation of colorectal cancer cell lines. **A, B:** STR profiles of the colon cancer cell line HCT116 (**A**) and SW480 (**B**). **C:** Signals of CD19 (Green) were detected with flow cytometry in Raji cell, but not in CRC cell lines.
