# Supplementary material for: IgG Expression in Human Colorectal Cancer and Its Relationship to Cancer Cell Behaviors
Source: PLoS One. 2012 Nov 1;7(11):e47362. doi: 10.1371/journal.pone.0047362 (PMC3486799; doi:10.1371/journal.pone.0047362)
Supplement: Figure S3 — Reduction of IgG expression by siRNA affects the biologic behaviors of LOVO cells. A: Effect of IgG down-regulation. B: MTS assay shows that IgG down-regulation inhibits growth of LOVO cells. C: AnnexinV-PI Immunofluorescence double staining analysis shows that the apoptotic LOVO cells when IgG was downregulation, nuclear staining with DAPI. D: Invasion assay was carried out with transwell in 24 wells when si-IgG transfected into LOVO cells for 24 hours. (PDF) [file pone.0047362.s003.pdf]

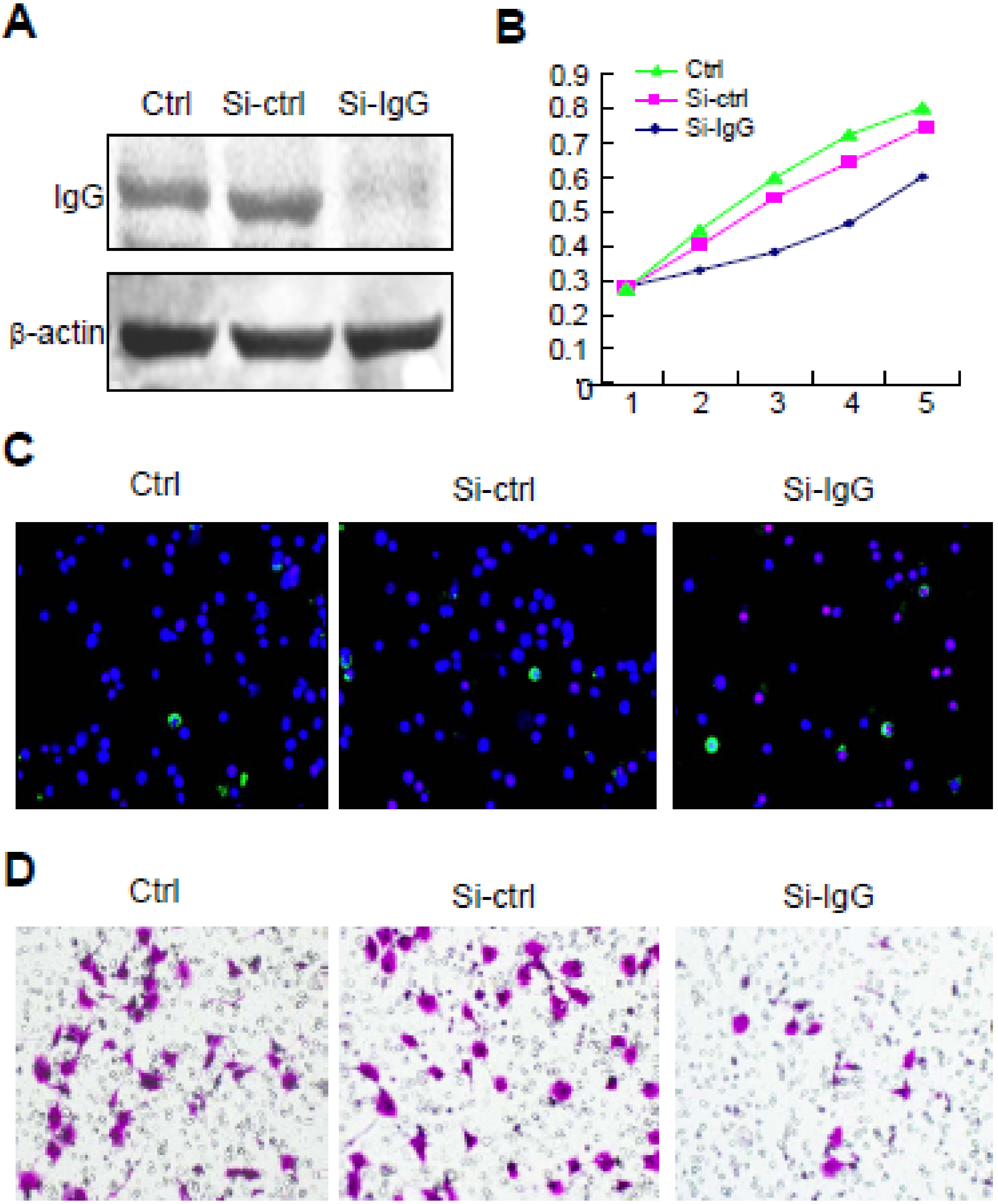

Figure S3. Reduction of IgG expression by siRNA affects the biologic behaviors of LOVO cells. **A:** Effect of IgG down-regulation. **B:** MTS assay shows that IgG down-regulation inhibits growth of LOVO cells. **C:** AnnexinV-PI Immunofluorescence double staining analysis shows that the apoptotic LOVO cells when IgG was downregulation, nuclear staining with DAPI. **D:** Invasion assay was carried out with transwell in 24 wells when si-IgG transfected into LOVO cells for 24 hours.
