# Supplementary material for: IgG Expression in Human Colorectal Cancer and Its Relationship to Cancer Cell Behaviors
Source: PLoS One. 2012 Nov 1;7(11):e47362. doi: 10.1371/journal.pone.0047362 (PMC3486799; doi:10.1371/journal.pone.0047362)
Supplement: Table S1 — Primary Antibody Details. (PDF) [file pone.0047362.s004.pdf]

Table S1. Primary Antibody Details

Primary antibody	Source	Dilution	Incubation	Supplier
IgG (γ chain specific)	Rabbit	1:200	4℃, overnight	Dako, Glostrup, Denmark
Immunoglobulin γ chain	Mouse	1:1000	4℃, overnight	Sigma-Aldrich, St Louis, MO, USA
Immunoglobulin κ chain	Mouse	1:1000	4℃, overnight	Abcam, Cambridge, MA, USA
CEA	Mouse	1:500	4℃, overnight	Sigma-Aldrich, St Louis, MO, USA
CD16	Mouse	1:25	4, overnight℃	Santa Cruz, Santa Cruz, CA, USA
CD32	Mouse	1:50	4℃, overnight	Santa Cruz, Santa Cruz, CA, USA
CD64	Mouse	1:50	4℃, overnight	Santa Cruz, Santa Cruz, CA, USA
P53	Mouse	1:200	4℃, overnight	Zymed Laboratories, San Francisco, CA, USA
PCNA	Mouse	1:2000	4℃, overnight	Cell Signaling, Danvers, MA, USA
Bcl-2	Mouse	1:50	4℃, overnight	Abcam, Cambridge, MA, USA
MMP-2	Mouse	1:50	4℃, overnight	Millipore, Bilerica, MA, USA
NF-κB	Rabbit	1:50	4℃, overnight	Cell Signaling, Danvers, MA, USA
Cyclin D1	Rabbit	1:25	4℃, overnight	Cell Signaling, Danvers, MA, USA
