# Supplementary material for: IgG Expression in Human Colorectal Cancer and Its Relationship to Cancer Cell Behaviors
Source: PLoS One. 2012 Nov 1;7(11):e47362. doi: 10.1371/journal.pone.0047362 (PMC3486799; doi:10.1371/journal.pone.0047362)
Supplement: Table S2 — Primers for PCR Amplification. (PDF) [file pone.0047362.s005.pdf]

Table S2. Primers for PCR Amplification

Gene name	Primer sequence 5'-3'	Annealing temperature	Product size (base pairs)
IGHG1	GACTGGCTGAATGGCAAGGAG (sense)<br>GGCGATGTCGCTGGGATAGAA (antisense)	56℃	201
CDR3	External V3, GAGGTGCAGCTCGAGCAGTCAGG (sense)<br>V4f, CAGGTGCAGCTGCTCGAGTCGGG (sense)<br>V6, CAGGTACAGCTCGAGCAGTCAGG (sense)<br>LJH, TGAGGAGACGGTGACC (antisense)<br>Internal ACACGGCYSTGTATTACTGT(sense)<br>GTGACCAGGGTNCCTTGGCCCCAAG(antisense)	Touch down from 62℃ to 52℃<br>55℃	80-110
Igκ	TGAGCAAAGCAGACTACGAGA (sense)<br>GGGGTGAGGTGAAAGATGAG (antisense)	54℃	231
AID	External GAAGAGGCGTGACAGTGCT (sense)<br>CGAAATGCGTCTCGTAAGT (antisense)<br>Internal CCTTTTCACTGGACTTTGG (sense)<br>TGATGGCTATTTGCACCCC (antisense)	54℃<br>52℃	294
RAG1	External TGGATCTTTACCTGAAGATG (sense)<br>CTTGGCTTTCCAGAGAGTCC (antisense)<br>Internal CACAGCGTTTTGCTGAGCTC (sense)<br>AGCTTGCCTGAGGGTTCATG (antisense)	52℃<br>54℃	327
RAG2	External TGGAAGCAACATGGGAAATG (sense)<br>CATCATCTTCATTATAGGTGTC (antisense)<br>Internal TTCTTGGCATACCAGGAGAC (sense)<br>CTATTTGCTTCTGCACTG (antisense)	52℃<br>52℃	193
CD19	TACTATGGCACTGGCTGCTG (sense)<br>CACGTTCCCGTACTGGTTCT (antisense)	54℃	218
Vκ	GACATCGAGCTCACCCAGTCTCC (sense)<br>GAAATTGAGCTCACGCAGTCTCCA (sense)<br>ACGTTTGAATTCCACCTTGGTCCC (antisense)	54℃	280-295
Iγ- Cγ	External GGGCTTCCAAGCCAACAGGGCAGGACA (sense)<br>CAAGCTGCTGGAGGGCACGGT (antisense)<br>Internal GGTGAACCGAGGGGCTTGT (sense)<br>CGCTGCTGAGGGAGTAGAGT (antisense)	54℃<br>52℃	332
Igλ	GAGCCTGACGCCTGAG (sense)<br>ATTGAGGGTTTATTGAGTGCAG (antisense)	54℃	220
β-actin	TAAAGACCTCTATGCCAACACAG (sense)<br>CACGATGGAGGGGCCGGACTCATC (antisense)	54℃	218
